# Pro-neuropeptide Y as a circulating biomarker for poor prognosis in prostate cancer

**DOI:** 10.1038/s41598-026-58517-8

**Published:** 2026-06-23

**Authors:** Erik Djusberg, Kristina Lundquist, Marie Lundholm, Andreas Josefsson, Camilla Thellenberg Karlsson, Jochen M. Schwenk, Maria Brattsand, Anders Bergh, Pernilla Wikström

**Affiliations:** 1 Department of Medical Biosciences, Pathology, Umeå University, Umeå, Sweden; 2 Department of Chemistry, Umeå University, Umeå, Sweden; 3 Department of Diagnostics and Intervention, Oncology, Umeå University, Umeå, Sweden; 4 Department of Diagnostics and Intervention, Urology and Andrology, Umeå University, Umeå, Sweden; 5 Science for Life Laboratory, Department of Protein Science, KTH Royal Institute of Technology, Solna, Sweden

**Keywords:** Neuropeptide Y, Pro-NPY, Prostate cancer, Plasma, Prognosis, Biomarkers, Cancer, Oncology

## Abstract

Prostate cancer (PCa) is common world-wide. Current diagnostics based on testing for circulating levels of prostate specific antigen (PSA) is unspecific, and novel prognostic markers are needed for personalized therapeutic strategies. Pro-neuropeptide Y (pro-NPY) has been reported as a tissue marker for PCa related to poor prognosis. This study explored the prognostic value of circulating pro-NPY in PCa. Plasma samples were obtained from two patient cohorts: (1) men examined due to increased PSA levels in 2003–2011 (n = 796) and (2) patients treated for PCa in 2013–2016 (n = 92). Cohort 2 also provided plasma samples collected ~ 3 months after therapy. For plasma pro-NPY assessment, a sandwich immunoassay was developed. In cohort 1, 315 patients were diagnosed with PCa at the time for blood sampling, 137 were diagnosed during follow-up, and 344 remained disease-free. Plasma pro-NPY provided independent prognostic information from PSA regarding time to metastasis and PCa death. In cohort 2, high plasma pro-NPY levels were confirmed associated with metastatic disease and poor survival. Plasma pro-NPY levels were normalized after androgen-deprivation therapy, suggesting androgen-regulation. In conclusion, high circulating pro-NPY levels are associated with metastasis and poor outcome in PCa. Prospective validation is needed before suggesting pro-NPY for clinical use. The underlying biology and consequences of pro-NPY overexpression remain to be understood.

**Supplementary Information:**

The online version contains supplementary material available at 10.1038/s41598-026-58517-8.

## Introduction

Prostate cancer (PCa) is the second most common cancer in men world-wide^1^. Early detection of PCa relies on testing for circulating prostate specific antigen (PSA) followed by histologic examination of image-guided biopsies^2^. Magnetic resonance imaging (MRI) for guidance of biopsies has reduced detection of clinically insignificant tumors, yet the low specificity of PSA still leads to treatment of indolent tumors^3^. Despite this overtreatment, up to one third of patients develop metastatic disease^4,5^, highlighting the need for novel prognostic markers to guide treatment intensity.

Pro-neuropeptide Y (pro-NPY) has been reported as a tissue marker of poor prognosis in PCa^6^ and has also been detected in patient plasma^7^. While a recent study reported limited added diagnostic value of plasma pro-NPY over PSA^8^, its prognostic potential remains unexplored. Pro-NPY is activated by proteolytic cleavage, and NPY acts via receptors Y1, Y2, Y4, and Y5 (Figure S1)^9^. Physiologically, NPY regulates blood pressure, feeding, and stress resilience^10,11^, and dysregulation is linked to psychiatric, metabolic, and oncological conditions^12–14^. In cancer, NPY promotes hallmarks such as proliferation, migration, inflammation, metabolism, angiogenesis and treatment resistance^15–19^.

In this study, two independent patient cohorts were used to retrospectively explore plasma pro-NPY as a prognostic biomarker in PCa. A sandwich immunoassay (SIA) was developed to quantify plasma pro-NPY, which was analyzed pre-treatment in relation to clinicopathological variables, time to metastasis, and death. In one cohort, levels were also assessed ~ 3 months after treatment initiation.

## Materials and methods

### Patients and samples

In cohort 1 (Tables S1–S2), plasma samples were consecutively collected from patients examined for suspected PCa at the Urology Clinic in Umeå (2003–2011). Patients diagnosed within 6 months of sampling were classified as having PCa at sampling, and those diagnosed later as PCa during follow-up. Samples were obtained from 315 patients diagnosed at sampling, 137 diagnosed later, and 344 who remained disease-free. Patients received treatments according to Swedish national risk-group guidelines at the time; conservative, radical prostatectomy (RP), radiation therapy (RT), neoadjuvant androgen-deprivation therapy (ADT) before RT, or continuous ADT (Table S2).

In cohort 2 (Table S3), plasma samples were collected within the Uppsala-Umeå Comprehensive Cancer Consortium (U-CAN) from 92 patients at diagnosis and ~ 3 months after initiation of primary treatment; RP, neoadjuvant ADT before RT, or continuous ADT + /− add-on treatment with abiraterone or docetaxel within 6 months (Umeå, 2013–2016) (Table S3).

Whole blood was collected in EDTA tubes, centrifuged (1500 × g for 15 min, cohort 1; 2400 × g for 7 min, cohort 2), aliquoted, and stored at − 80 °C, with processing time not exceeding 1 h for cohort 1 and 4 h for cohort 2. Patient and tumor characteristics at diagnosis were retrieved from the National Prostate Cancer Register (2017). Final follow-up occurred in 2020 (cohort 1) and 2025 (cohort 2). Time was measured from blood sampling to metastasis, PCa-specific death, or death from any cause.

The study was approved by the local ethics review board of Umeå University (Dnr 2013–57-31 M with amendment Dnr 2017–197-32 M). All patients gave their informed consent, and the study was conducted in accordance with the Declaration of Helsinki.

### Sandwich immunoassay (SIA)

A SIA for quantification of pro-NPY levels in plasma was developed by the Affinity Proteomics Unit at Science for Life Laboratory (Stockholm). Initially, nine anti-NPY antibodies were tested in combination for suitable assay pairs. Specificity was evaluated by mass spectrometry^20^ (data not shown).

Following an established protocol^21^, a pro-NPY-specific antibody (HPA045572, Atlas Antibodies, Stockholm, Sweden) (Fig. S1) was coupled to color-coded MagPlex beads (Luminex, Austin, TX, US) to capture pro-NPY from plasma. Samples were diluted 1:10 in 50 µl LowCross assay buffer (Candor Bioscience GmbH, Wangen im Allgäu, Germany) and randomized across 384-well assay plates. Rabbit IgG and bare bead controls were added per well to detect unspecific binding, and blank wells were evenly distributed across the assay plates to determine background. Samples were incubated overnight at RT with shaking (650 rpm), washed 3 × in PBS-T (BioTek EL405), before incubated for 90 min with a biotinylated anti-NPY antibody (SAB1404138, 2 µg/ml, Sigma-Aldrich, Saint Louis, MO, US) (Fig. S1). After 3 additional washes, immunocomplexes were fixed with 0.4% PFA, followed by incubation with R-PE–labeled streptavidin (1:750, 30 min). After washing 3 × in PBT-S, 100 µl were added to each well for readout. Median fluorescence intensities (MFI) were read (≥ 32 beads/sample) on a FlexMap3D instrument (Luminex Corp). Pro-NPY concentrations were calculated by comparing MFI values to a standard curve generated from recombinant pro-NPY (100 ng/ml–1 pg/ml), using a 5-parameter logistic fit (LL.5 in *drm*, *drc* R package).

### Statistics

Continuous variables were compared by the Mann–Whitney U-test or the Wilcoxon paired-test, and categorical variables by the Chi-square test. Survival was compared using the log-rank test or Cox regression analysis with PCa diagnosis, metastasis diagnosis, or death as events. Receiver operating characteristics (ROC) curve analyses of plasma pro-NPY and serum PSA assessed diagnostic and prognostic performance within 10 years of blood sampling, including only patients with sufficient follow-up. Optimal cutoff values were determined from calculating the Youden’s index. Mann–Whitney U test was performed to determine if the area under curve (AUC) was significantly different from 0.5. Delong’s test in R (v4.3.2; R Core Team 2023) was applied to compare AUCs between models. In Kaplan–Meier analysis, cut-offs for pro-NPY levels were explored based on quartiles (Q1–4) and percentiles. Cox regression and ROC analyses were performed based on log10-transformed pro-NPY and PSA, to obtain more normally distributed data. Correlations between non-normally distributed variables were evaluated using Spearman’s rank test (IBM SPSS Statistics, v. 28.0.0.1).

## Results

### Assay development for plasma pro-NPY

To quantify pro-NPY in plasma, a SIA was developed in collaboration with the SciLifeLab affinity proteomics platform. The optimized assay, using an anti–pro-NPY capture antibody and an anti-NPY detection antibody (Fig. S1), was validated to detect pro-NPY exclusively and demonstrated a dynamic range of 1 pg/ml–100 ng/ml. Using this method, pro-NPY levels were measured in plasma samples (n = 964) from two patient cohorts with a coefficient of variation < 10%. The lower limits of quantification (LLOQ) were 0.9 pg/ml for cohort 1 and 27 pg/ml for cohort 2. Samples below the LLOQ (7% in cohort 1 and 14% in cohort 2) were assigned the arbitrary lowest levels per cohort.

### High plasma Pro-NPY levels in patients with poor outcome

**Fig. 1:**
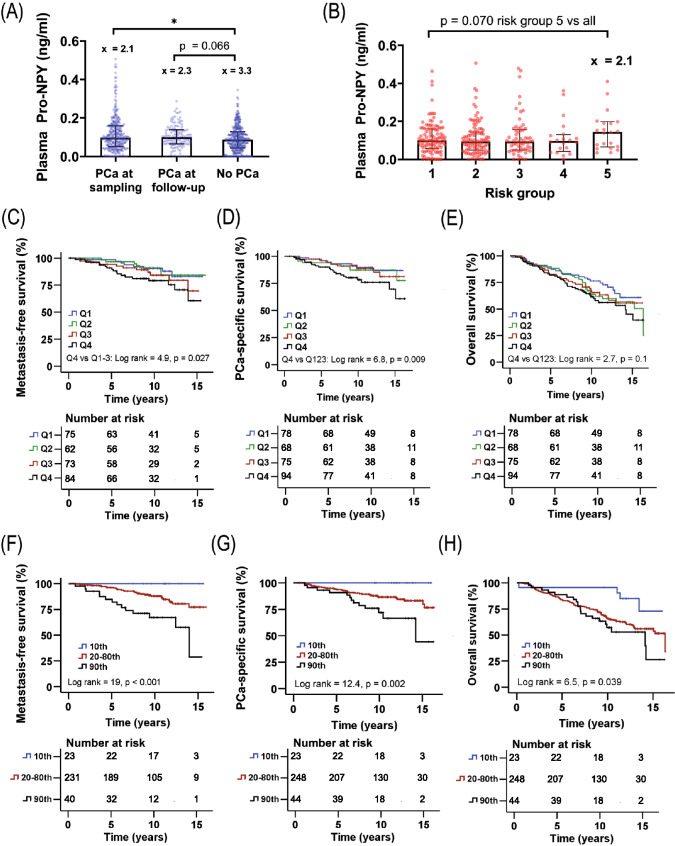
Circulating pro-NPY levels are increased in prostate cancer (PCa) patients and prognostic for metastatic disease and poor survival. (**A**) Plasma pro-NPY levels in patients diagnosed at time for blood sampling (n = 315), at later time-points (n = 137), and in those who remained disease-free during follow-up (n = 344). (**B**) Plasma pro-NPY levels in patients diagnosed at sampling in relation to risk group stratification based on Gleason score, TNM stage, and serum PSA (Table S1-S2). Bars show median and inter-quartile range with individual values plotted. *P < 0.05 according to Mann–Whitney U test. x = outlier value (ng/ml). (**C**–**H**) Kaplan–Meier analysis of plasma pro-NPY levels in patients diagnosed at sampling in relation to metastasis-free survival (**C**, **F**), PCa-specific survival (**D**, **G**), and overall survival (**E**, **H**). The pro-NPY levels were analyzed in quartiles (Q1-Q4) in **C-E** and in 3 groups cut by the 10^th^ and 90^th^ percentiles in **F**–**H**, based on all samples in the cohort (n = 796). Patients with metastasis diagnosis at the time for blood sampling were excluded in analysis of metastasis-free survival (**C**, **F**).

Plasma pro-NPY levels were measured in cohort 1, comprising of men investigated for suspected PCa due to elevated PSA. Patients were diagnosed with PCa at blood sampling (n = 315), diagnosed later (n = 137), or remained disease-free during follow-up (n = 344; median 11 years, IQR 9–14) (Table S1). As shown in Fig. 1, pro-NPY levels varied widely across all groups, although patients diagnosed with PCa at sampling had significantly higher levels than those who remained disease-free (median 0.0970 vs. 0.0872 ng/ml, p = 0.007) (Fig. 1A).

In patients diagnosed with PCa at blood sampling, pro-NPY levels were assessed in relation to clinical variables at diagnosis (Table S2). Patients in risk group 5 (M1 disease or PSA > 100 µg/l) showed a trend toward higher pro-NPY levels compared with all other risk groups combined (median 0.145 vs. 0.0965 ng/ml, p = 0.070) (Fig. 1B). No significant associations were observed between pro-NPY and Gleason score, T, N, or M stage, or plasma PSA (Fig. S2). Pro-NPY levels were also unrelated to patient age (Sr = 0.069, p = 0.23) but showed weak inverse correlation to sample storage time (Sr = –0.124, p < 0.001).

For prognostic evaluations, pro-NPY levels at blood sampling were analyzed in relation to time to metastasis and death, independent of primary treatments. Patients in cohort 1 were stratified into Q1-4 for Kaplan–Meier analysis (median 0.0917 ng/ml, IQR 0.0519;0.144, n = 796). Among men diagnosed with PCa at the time for blood sampling, high pro-NPY levels (Q4) were associated with short metastasis-free survival (Q4 vs. Q1-3, p = 0.027, M1 cases excluded) (Fig. 1C) and short PCa-specific survival (Q4 vs. Q1-3, p = 0.009) (Fig. 1D), but not overall survival (Fig. 1E). Patients in the 90^th^ percentile (≥ 0.199 ng/ml) had the poorest outcomes, whereas no metastases or PCa deaths were observed in the 10th percentile (< 0.0142 ng/ml) (Fig. 1F, G), likely contributing to the observed difference in OS (Fig. 1H). Sensitivity analysis was performed by removing samples with pro-NPY levels below the LLOQ (n = 56) and recalculating Q1-4 and percentiles. The results were generally consistent with those shown in Fig. 1, with the relatively highest pro-NPY levels being associated with the poorest prognosis (Fig. S9). Naturally, the Q1 and the 10th percentile in Fig. S9 consisted of higher pro-NPY levels than their counterparts in Fig. 1, which likely explains the small differences observed and the absence of a favorable prognosis for the 10th percentile in Fig. S9 D–F.

In men diagnosed with PCa during follow-up (median time to diagnosis 4.2 years, IQR 2.1–6.9), low pro-NPY (Q1) was associated with longer time to diagnosis (Q1 vs. Q2–4, p = 0.028) (Fig. S3A). Higher levels (Q3–4) predicted shorter metastasis-free survival (p = 0.040) and showed a trend toward shorter PCa-specific survival in Q4 (p = 0.054), with no association to overall survival (Fig. S3B-D). Analyses based on the 10th and 90th percentiles were inconclusive in this patient group plausibly due to small subgroup sizes (data not shown).

Pro-NPY levels were not associated with risk-group classification at later diagnosis (GS, TNM stage, PSA) (Fig. S4) or age (Sr = 0.10, p = 0.23). Among men without PCa, pro-NPY showed no relationship with age (Sr = 0.040, p = 0.46), PSA, or overall survival (Fig. S5).

### Increased prognostic value by combined evaluation of plasma pro-NPY and PSA

In patients diagnosed with PCa at blood sampling, plasma pro-NPY provided prognostic information beyond current clinical risk markers. In multivariate Cox models, higher pro-NPY was associated with increased risk of metastasis (HR 2.7, CI 1.1–6.4, p < 0.05; M1 excluded) and PCa-specific death (HR 3.3, CI 1.4–7.4, p < 0.01), independent of PSA levels, patient age, and plasma sample storage time (Table 1). Pro-NPY also added independent value to age and risk groups 1–4 for predicting metastasis (HR 3.0, CI 1.3–7.1, p < 0.05), but not PCa-specific mortality (Table 1).

**Table 1:** Multivariate cox regression analysis of plasma pro-NPY levels in combination with clinical variables at diagnosis in relation to time to metastasis and death from prostate cancer (PCa).

Metastasis^1^				PCa-death			
Variable	N	HR	95% CI for HR	Variable	n	HR	95% CI for HR
Pro-NPY	290	2.7*	1.1–6.4	Pro-NPY	311	3.3**	1.4–7.4
PSA	290	4.2**	1.6–11	PSA	311	4.9***	3.3–7.1
Age	290	1.076**	1.03–1.13	Age	311	1.064**	1.014–1.12
Plasma storage time	290	1.005	0.87–1.16	Plasma storage time	311	1.11	0.96–1.1,3

Variable	N	HR	95% CI for HR	Variable	n	HR	95% CI for HR
Pro-NPY	281	3.0*	1.3–7.1	Pro-NPY	304	1.9	0.94–3.7
Age	281	1.060*	1.009–1.12	Age	304	1.050*	1.001–1.10
Risk group				Risk group			
1	104	Ref		1	104	Ref	
2	99	3.9*	1.3–12	2	99	5.4*	1.17–25
3	62	4.6*	1.4–15	3	62	6.2*	1.3–29
4	19	18***	5.2–61	4	20	27***	5.6–130
5	Excluded	-	-	5	22	120***	25–540
Plasma storage time	281	1.023	0.89–1.18	Plasma storage time	304	1.15	0.99–1.33

Cox regression analysis with pro-NPY, PSA, age and storage time used as continuous variables, and risk group as a categorical variable. Log 10 of Pro-NPY and PSA were used. The models include patients in cohort 1 diagnosed at blood sampling. ^1^Patients with metastasis diagnosis at the time for blood sampling were excluded in model for metastasis-free survival. *p < 0.05, **p < 0.01, ***p < 0.001.

HR hazard ratio, CI confidence interval, Ref reference category.

**Fig. 2:**
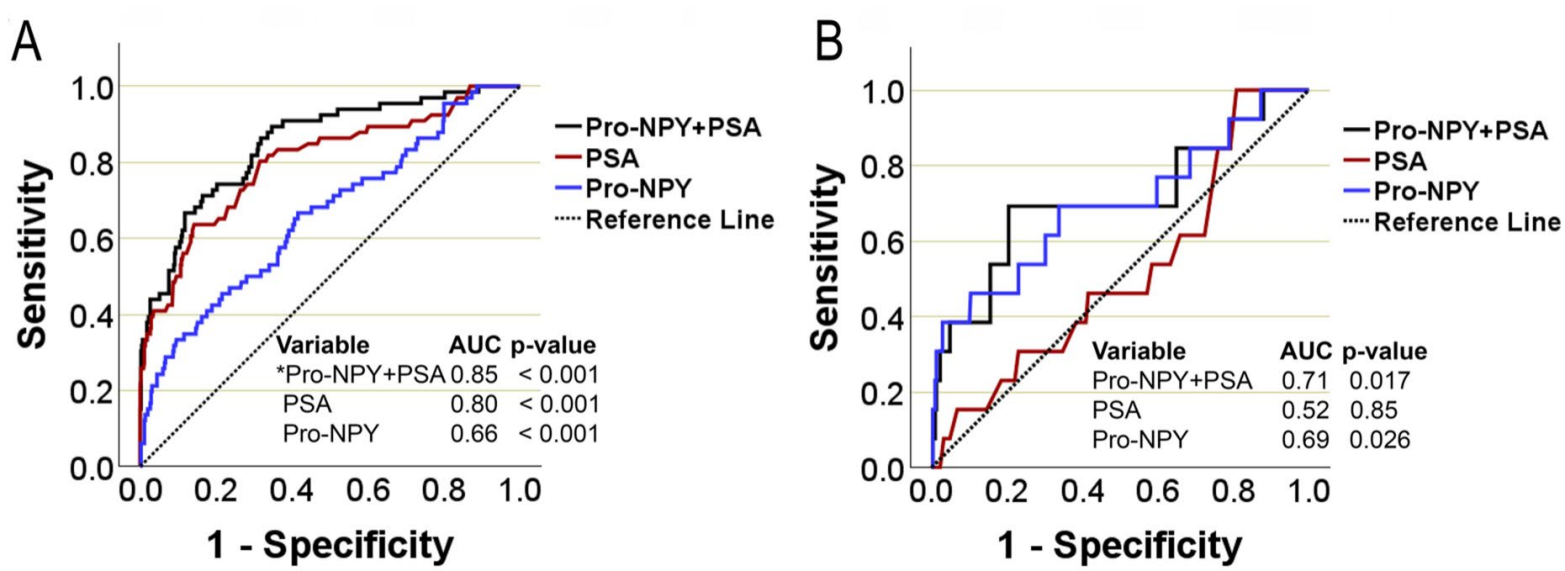
Plasma pro-NPY independently and in combination with serum PSA predicts metastasis within 10 years from blood sampling. ROC analysis of plasma pro-NPY (blue), PSA (red) and both markers combined (black) in relation to their ability to predict metastasis within 10 years in all patients with adequate follow-up (**A**, 66 events among 517 patients) and in cases with PSA levels < 10 µg/l (**B**, 13 events among 321 patients). Combined models were created using binary logistic regression and graphs constructed with logarithmized dependent variables (log10). Mann–Whitney U test was used to check if model was significantly different from the reference line. Delong’s test was applied to compare the AUC of different models. *P = 0.011 when comparing the combined model with the PSA model.

ROC analyses of all men in cohort 1 evaluated circulating pro-NPY and PSA as single markers and in combination for predicting PCa diagnosis, metastasis, and PCa-specific death within 10 years. As shown in Fig. 2A, pro-NPY and PSA individually predicted metastasis (AUC 0.66 and 0.80, respectively; p < 0.001), and the combined model improved performance (AUC 0.85, p < 0.001), outperforming PSA alone (p = 0.011). Sensitivity analysis performed after removing samples with pro-NPY levels below the LLOQ (n = 56) (Fig. S9 G-H) gave results comparable to results in Fig. 2. When applying the optimal cut-off value according to Youden’s index, the combined model yielded the following metrics: 97 patients above the cut-off, with a sensitivity of 0.67 and specificity of 0.88. In comparison, the PSA-only model identified 106 patients above the cut-off, with a sensitivity of 0.64 and specificity of 0.86. Thus, the combined model resulted in 9 less patients (19% vs. 21%) classified as high risk with a slightly higher sensitivity (44 vs. 42 of 66 metastases correctly predicted). Pro-NPY and PSA, individually or combined, also predicted PCa diagnosis and PCa-specific death, although combined models were not significantly better than PSA alone (Fig. S6A–B).

In men with PSA < 10 µg/l, pro-NPY predicted metastasis (AUC 0.69, p = 0.026), as did the combined model (AUC 0.71, p = 0.017), while PSA alone did not (Fig. 2B). Within this low PSA range, the optimal cut-off for the combined model gave sensitivity 0.69 and specificity 0.80. Nonetheless, PSA retained predictive value for PCa diagnosis and PCa-specific death in this subgroup (Fig. S6C–D).

### Validation of high plasma pro-NPY as a marker for poor outcome, and its normalization by androgen-deprivation therapy

**Fig. 3:**
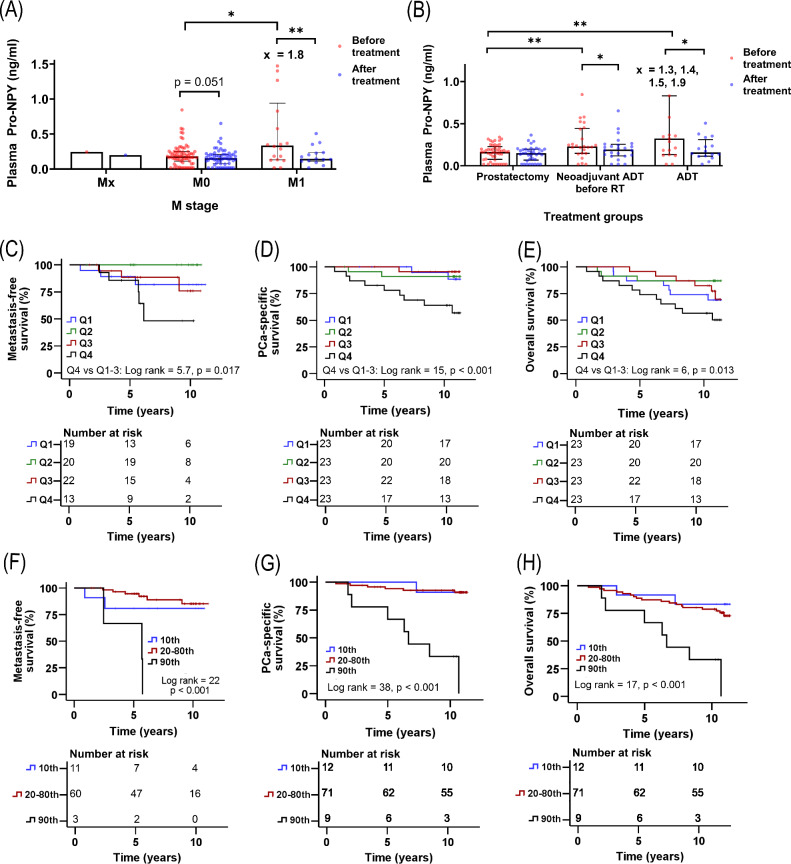
Validation of high plasma pro-NPY levels in patients with poor prognosis and normalization of pro-NPY after androgen-deprivation therapy. (**A**) Plasma pro-NPY levels before and about 3 months after primary treatment in relation to M stage. (**B**) Plasma pro-NPY levels before and about 3 months after initiation of primary treatments. Bars show median and inter-quartile range with individual values plotted. *p < 0.05, **p < 0.01, according to Mann–Whitney U test comparing pre-treatment levels and Wilcoxon paired-test for pre- vs. post-treatment comparisons. x = outlier values (ng/ml). (**C**–**H**) Kaplan Meier analysis of plasma pro-NPY levels before treatment in relation to metastasis-free survival (**C**, **F**), PCa-specific survival (**D**, **G**) and overall survival (**E, H**). Patients were divided into quartiles (Q1–Q4) in (**C**–**E**) and in 3 groups based on the 10^th^ and 90^th^ percentiles in (**F**–**H**) based on all pre-treatment pro-NPY samples analysed in the cohort (n = 92). Patients with metastasis diagnosis at the time for blood sampling were excluded in analysis of metastasis-free survival (**C**, **F**).

To validate the prognostic value of circulating pro-NPY, plasma levels were quantified in an independent cohort (cohort 2) with samples collected shortly before and about 3 months after initiation of primary treatment (Table S3). At baseline, M1 patients had significantly higher pro-NPY levels than non-metastatic cases (median 0.346 vs. 0.186 ng/ml, p = 0.016), and these levels normalized after treatment (Fig. 3A). Consistently, patients receiving neoadjuvant ADT plus RT or continuous ADT showed elevated pre-treatment pro-NPY that normalized after therapy, whereas levels in men treated with radical prostatectomy were lower pre-treatment and remained unchanged (Fig. 3B). The reduction in pro-NPY levels after ADT varied markedly between patients, with large decreases observed in M1 patients with high pre-therapy levels (Fig. S7). Higher pre-treatment pro-NPY levels were associated with higher GS, T stage, risk group, and PSA (Fig. S8), with no association to N1 stage, age (Sr = –0.064, p = 0.55), or body mass index (Sr = –0.055, p = 0.71).

For survival analyses, patients were stratified into Q1-4 based on baseline pro-NPY levels (median 0.191 ng/ml, IQR 0.127–0.300). High pro-NPY (Q4) was associated with short metastasis-free (p = 0.017), PCa-specific (p < 0.001), and overall survival (p = 0.013) (Fig. 3C–E). As in cohort 1, pro-NPY levels in the 90th percentile (≥ 0.557 ng/ml) were associated to the poorest outcomes across all endpoints (p < 0.001), while other groups experienced few events (Fig. 3F–H). As in cohort 1, sensitivity analysis excluding samples with pro-NPY levels below the LLOQ (n = 13) and recalculating Q1–Q4 percentiles in cohort 2 showed that patients with the highest pro-NPY levels had the worst outcomes (Fig. S10).

## Discussion

This study demonstrates relatively higher plasma pro-NPY levels in PCa patients with particularly poor clinical outcomes. By quantifying pre-treatment levels of plasma pro-NPY, we show that patients with the highest levels experience disproportionately high rates of adverse PCa events (metastasis development and PCa-specific mortality), a finding consistent across two cohorts. These results indicate circulating pro-NPY as a potential prognostic biomarker for PCa, providing novel insight beyond its diagnostic evaluation in previous studies^7,8^.

We observed slightly higher circulating pro-NPY levels in men diagnosed with PCa compared to men with elevated PSA who remained tumor-free for over ten years. Lower pre-diagnostic pro-NPY was associated with reduced risk of later PCa, but combining pro-NPY with PSA did not significantly improve diagnostic prediction, consistent with Maurer et al.^8^. In contrast, pro-NPY provided independent prognostic information beyond PSA.

Relatively higher pro-NPY levels were associated with increased risk of metastasis and PCa-specific death in both cohorts examined. Notably, patients in the extremes of the pro-NPY range exhibited strikingly different clinical courses, with those in the 90th percentile having particularly unfavorable outcomes, whereas those in the 10th percentile rarely experienced any PCa-related events. Multivariate Cox models confirmed that plasma pro-NPY predicted metastasis independently of GS, TNM stage, and PSA, suggesting added clinical utility.

Patients classified as low- or intermediate-risk are typically managed conservatively or treated with curative intent via surgery or radiotherapy. In retrospect, patients in our cohorts with very high pro-NPY might have benefited from high-risk classification and intensified therapy, whereas low levels could have supported conservative management. Overall, circulating pro-NPY may help identify aggressive PCa among patients with otherwise favorable profiles.

Regarding pro-NPY associations with clinicopathological factors, our results varied. In cohort 2, pro-NPY levels correlated positively with GS and TNM stage, consistent with earlier reports^7^, whereas such associations were less evident in cohort 1. A plausible explanation is that cohort 2 included a larger proportion of advanced tumors, more likely to influence plasma NPY levels. Differences in sample storage time and potential degradation of plasma pro-NPY levels over time may also have contributed, as median storage time was 12 and 3 years for cohort 1 and 2, respectively.

A recent study reported contrasting findings with higher circulating NPY levels observed in patients with localized PCa than in those with metastatic disease^22^. The reason for this discrepancy remains unclear. The specificity of the ELISA used in that study is uncertain, whereas we validated our antibodies using orthogonal mass spectrometry. In addition, clinical outcomes were not assessed. Notably, their study analyzed serum samples from fasting individuals, and it remains unclear to what extent feeding status contributed to the high variability in circulating pro-NPY levels observed in our cohort. In healthy individuals, plasma NPY levels decrease following high-carbohydrate intake, while patients with anorexia exhibit elevated plasma NPY levels^23^, suggesting a link between NPY signaling and nutritional status. Consistent with this, NPY signaling may also play a role in cancer cachexia, as animals with tissue-specific knockdown of NPY in peripheral sympathetic nerves exhibit reduced thermogenesis and increased weight^24^.

In our study, body weight data were available only for patients undergoing RP in cohort 2. Within this subcohort, plasma pro-NPY levels were not associated with body weight. However, these patients had significantly lower plasma pro-NPY levels than patients with locally advanced or metastatic tumors, preventing any conclusions regarding the relationship between elevated plasma pro-NPY and cancer cachexia in advanced disease. Addressing this question would require longitudinal monitoring of both NPY levels and body weight throughout disease progression.

Other factors that may influence pro-NPY variability include sympathetic activation, exemplified by a ~ twofold NPY increase within minutes in response to a minor physical challenge such as standing up^25^. Several pharmaceuticals and common conditions such as obesity, psychiatric disorders and cardiovascular disease also show increased levels of plasma NPY^13,14,16,25–28^. Taken together, this emphasizes the need for strictly standardized sampling protocols, and the consideration of individual-related conditions when interpreting plasma pro-NPY levels.

The high pre-treatment pro-NPY levels seen in patients with advanced disease, and their normalization about three months after ADT, indicate that tumor NPY expression is a significant source to the plasma pro-NPY levels in PCa patients. We hypothesize that tumor NPY expression is regulated by androgens, either directly through AR-regulated NPY expression or indirectly through AR-stimulated expression of the TMPRSS2-ERG fusion gene and subsequent ERG-driven NPY expression, as previously suggested^6,29–32^. Another plausible mechanism behind high tumor NPY expression could involve sympathetic nerve infiltration and norepinephrine secretion, known to enhance NPY production^17,28^. Perineural invasion, common in PCa, has been linked to both NPY expression and disease progression^17,33^. Also, hypoxia-induced NPY signaling has been demonstrated for endothelial cells and various types of cancers^34–36^.

Functional effects of tumor NPY overexpression remain unclear but may involve enhanced glycolysis, proliferation, migration, and survival^15–17,19,29^. These effects could be directly mediated by NPY receptors on tumor cells or indirectly through receptor positive cells in the tumor microenvironment^16–18,33^. Potentially, NPY signaling may be induced as a stress response to therapeutic interventions such as radiotherapy or chemotherapy, conferring a survival advantage^17,19^. Post-translational modifications of NPY could alter receptor affinity, shifting signaling from the growth-regulatory NPY1R toward the more oncogenic NPY2R/NPY5R axis associated with angiogenesis, therapy resistance, and cancer progression^34,35,37^.

Limitations of the study include lack of matched tumor samples for evaluating the correlation between tumor expression and plasma pro-NPY levels. Also, it remains unclear whether elevated plasma pro-NPY reflects increased levels of active NPY. The SIA method used exclusively quantified pro-NPY, which requires proteolytic processing to become biologically active. A MS–based approach, would have allowed evaluation of pro-NPY and its derivatives^25^. Additionally, sampling was performed before PSMA-PET was implemented as a staging tool, and biases in baseline characteristics are thus possible.

## Conclusions

High circulating pro-NPY levels are associated with metastasis and poor outcomes in prostate cancer (PCa). Patients with elevated pro-NPY levels have an increased risk of metastasis and PCa-specific mortality, and pro-NPY may provide prognostic information beyond prostate-specific antigen (PSA) and established risk factors. Measuring plasma pro-NPY might help identify aggressive tumors in men who otherwise present with favorable clinical profiles, such as low PSA levels. However, before pro-NPY can be recommended for clinical use, prospective studies are required to validate the added clinical value of measuring circulating pro-NPY in addition to PSA for risk stratification. For those studies, a robust and standardized assay must be developed, including consistent procedures for blood sampling. The underlying biology behind pro-NPY overexpression and its consequences also remain to be elucidated. Notably, reduced plasma pro-NPY levels following ADT suggest direct or indirect androgen regulation, indicating that pro-NPY may serve as a potential biomarker for predicting and monitoring treatment response.

## Supplementary Information

Supplementary Information 1.

Supplementary Information 2.

Supplementary Information 3.

Supplementary Information 4.

Supplementary Information 5.

Supplementary Information 6.

Supplementary Information 7.

Supplementary Information 8.

Supplementary Information 9.

Supplementary Information 10.

Supplementary Information 11.

Supplementary Information 12.

Supplementary Information 13.

## Data Availability

Data can be made available upon reasonable request.
