# Correction: Pro-neuropeptide Y as a circulating biomarker for poor prognosis in prostate cancer

**DOI:** 10.1038/s41598-026-61181-7

**Published:** 2026-07-15

**Authors:** Erik Djusberg, Kristina Lundquist, Marie Lundholm, Andreas Josefsson, Camilla Thellenberg Karlsson, Jochen M. Schwenk, Maria Brattsand, Anders Bergh, Pernilla Wikström

**Affiliations:** 1 Department of Medical Biosciences, Pathology, Umeå University, Umeå, Sweden; 2 Department of Chemistry, Umeå University, Umeå, Sweden; 3 Department of Diagnostics and Intervention, Oncology, Umeå University, Umeå, Sweden; 4 Department of Diagnostics and Intervention, Urology and Andrology, Umeå University, Umeå, Sweden; 5 Science for Life Laboratory, Department of Protein Science, KTH Royal Institute of Technology, Solna, Sweden

Correction to: *Scientific Reports* 10.1038/s41598-026-58517-8, published online 23 June 2026

The original version of this Article contains errors in Table 1, where one row of the table was omitted during production. Specifically, the 7th row that now reads: Variable N, HR, 95% CI was erroneously omitted.

The original table appears below:

**Table:** 

Metastasis1				PCa-death			
Variable	N	HR	95% CI	Variable	N	HR	95% CI
Pro-NPY	290	2.7*	1.1–6.4	Pro-NPY	311	3.3**	1.4–7.4
PSA	290	4.2**	1.6–11	PSA	311	4.9***	3.3–7.1
Age	290	1.076**	1.03–1.13	Age	311	1.064**	1.014–1.12
Plasma storage time	290	1.005	0.87–1.16	Plasma storage time	311	1.11	0.96–1.13
Pro-NPY	281	3*	1.3–7.1	Pro-NPY	304	1.9	0.94–3.7
Age	281	1.060*	1.009–1.12	Age	304	1.050*	1.001–1.10
Risk group				Risk group			
1	104	Ref		1	104	Ref	
2	99	3.9*	1.3–12	2	99	5.4*	1.17–25
3	62	4.6*	1.4–15	3	62	6.2*	1.3–29
4	19	18***	5.2–61	4	20	27***	5.6–130
5	Excluded	-	-	5	22	120***	25–540
Plasma storage time	281	1.023	0.89–1.18	Plasma storage time	304	1.15	0.99–1.33

The corrected table appears below:

**Table:** 

Metastasis1				PCa-death			
Variable	N	HR	95% CI	Variable	N	HR	95% CI
Pro-NPY	290	2.7*	1.1–6.4	Pro-NPY	311	3.3**	1.4–7.4
PSA	290	4.2**	1.6–11	PSA	311	4.9***	3.3–7.1
Age	290	1.076**	1.03–1.13	Age	311	1.064**	1.014–1.12
Plasma storage time	290	1.005	0.87–1.16	Plasma storage time	311	1.11	0.96–1.13

Variable	N	HR	95% CI	Variable	N	HR	95% CI
Pro-NPY	281	3*	1.3–7.1	Pro-NPY	304	1.9	0.94–3.7
Age	281	1.060*	1.009–1.12	Age	304	1.050*	1.001–1.10
Risk group				Risk group			
1	104	Ref		1	104	Ref	
2	99	3.9*	1.3–12	2	99	5.4*	1.17–25
3	62	4.6*	1.4–15	3	62	6.2*	1.3–29
4	19	18***	5.2–61	4	20	27***	5.6–130
5	Excluded	-	-	5	22	120***	25–540
Plasma storage time	281	1.023	0.89–1.18	Plasma storage time	304	1.15	0.99–1.33

The original article has been corrected.

