# Supplementary material for: Pro-neuropeptide Y as a circulating biomarker for poor prognosis in prostate cancer
Source: Sci Rep. 2026 Jun 23;16:19518. doi: 10.1038/s41598-026-58517-8 (PMC13291266; doi:10.1038/s41598-026-58517-8)
Supplement: Supplementary file 1 — Supplementary Information 1. [file 41598_2026_58517_MOESM1_ESM.pdf]

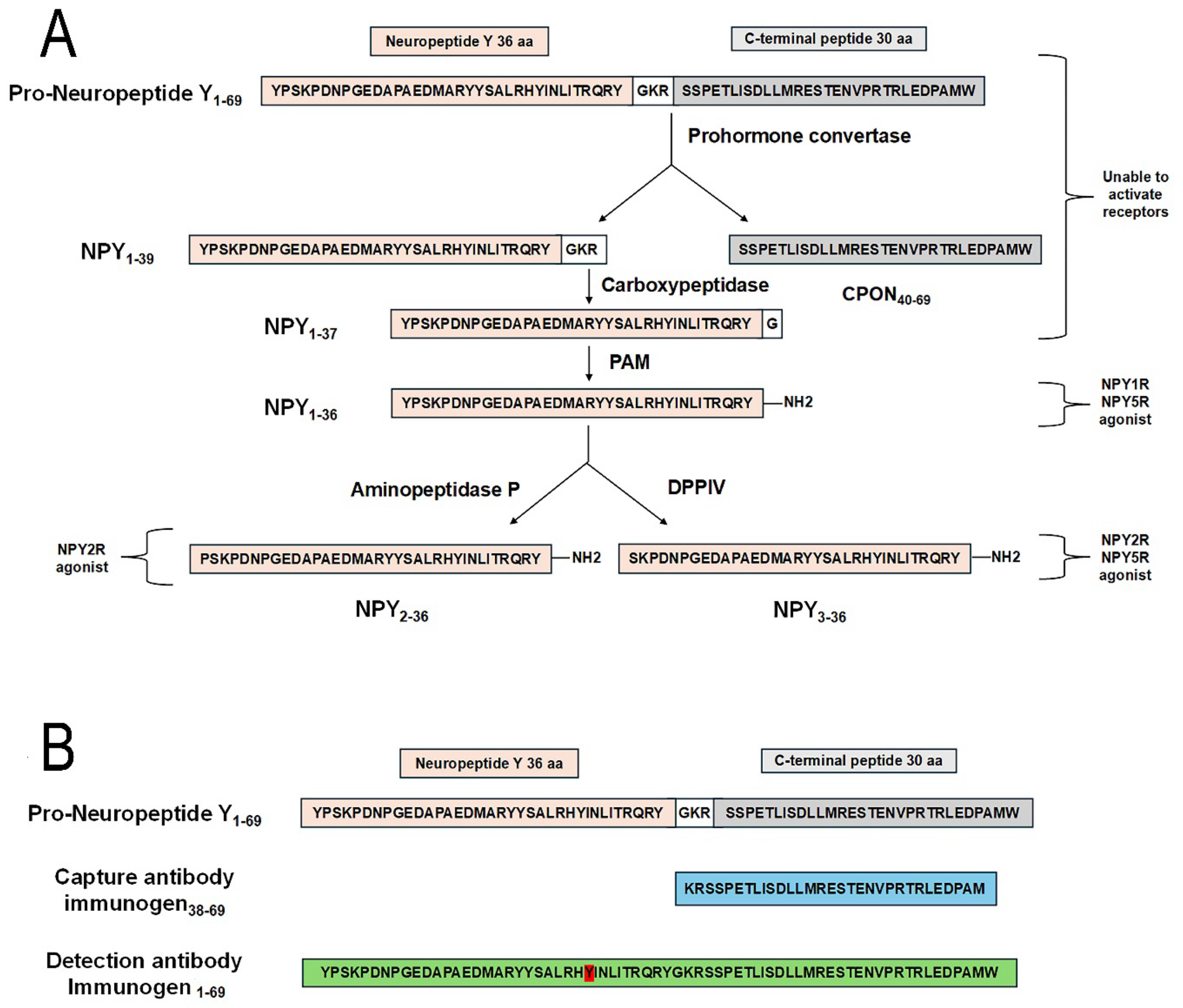

Notes: Y = GST-tagged tyrosine$_{27}$
Abbreviations: PAM; peptidylglycine α-amidating monooxygenase, DPPIV; Dipeptidyl peptidase IV, GST; Glutathione S-transferase

Fig. S1. A) The posttranslational processing of neuropeptide Y. Neuropeptide Y is translated into a pre-pro-peptide (not shown) that requires several proteolytic steps for activation. The signal peptide is removed, producing pro-NPY$_{1\text{-}69}$, which is sent through the secretory pathway and stored in secretory vesicles. Prohormone convertases cut the C-terminal peptide of NPY (CPON), which is released. Before or after secretion, the isoform NPY$_{1\text{-}39}$ is processed by carboxypeptidases and peptidylglycine α-amidating monooxygenases to produce the biologically active form NPY$_{1\text{-}36}$. B) Specificity of the antibodies used to construct the pro-NPY sandwich immunoassay used in the study for plasma measurements.
