# Supplementary material for: Pro-neuropeptide Y as a circulating biomarker for poor prognosis in prostate cancer
Source: Sci Rep. 2026 Jun 23;16:19518. doi: 10.1038/s41598-026-58517-8 (PMC13291266; doi:10.1038/s41598-026-58517-8)
Supplement: Supplementary file 2 — Supplementary Information 2. [file 41598_2026_58517_MOESM2_ESM.pdf]

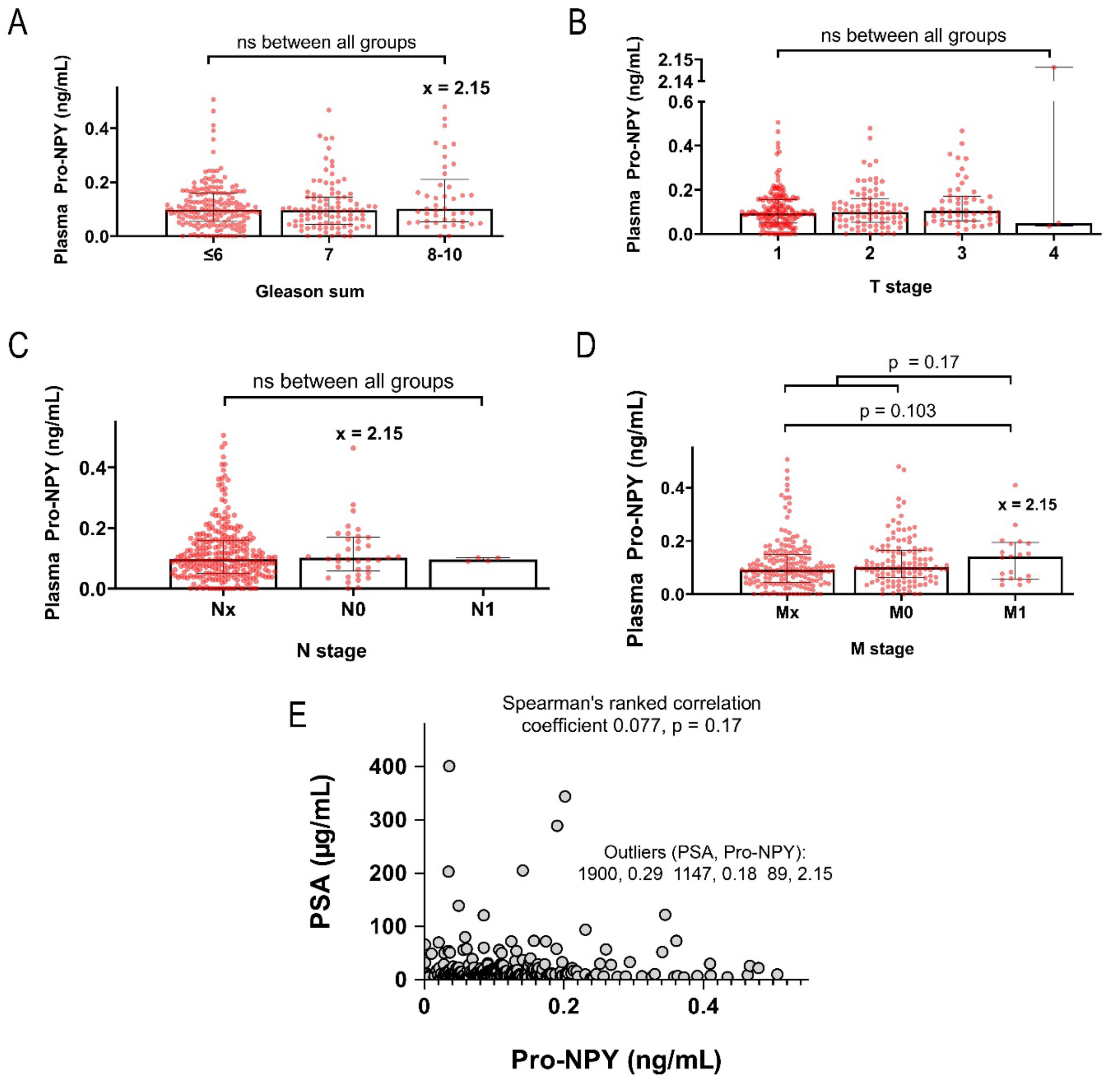

Fig. S2. Plasma pro-NPY levels in patients diagnosed with PCa at the time for blood sampling (n=315, cohort 1) in relation to Gleason score (A), T stage (B), N stage (C), M stage (D), and plasma PSA (E) at diagnosis (Table S1-2). Bars show median and inter-quartile range with individual values plotted. *$p < 0.05$, according to Mann-Whitney U test. x = outlier value (ng/ml). Bivariate correlation between plasma pro-NPY and serum PSA was analysed with Spearman's ranked correlation test.
