# Supplementary material for: Pro-neuropeptide Y as a circulating biomarker for poor prognosis in prostate cancer
Source: Sci Rep. 2026 Jun 23;16:19518. doi: 10.1038/s41598-026-58517-8 (PMC13291266; doi:10.1038/s41598-026-58517-8)
Supplement: Supplementary file 3 — Supplementary Information 3. [file 41598_2026_58517_MOESM3_ESM.pdf]

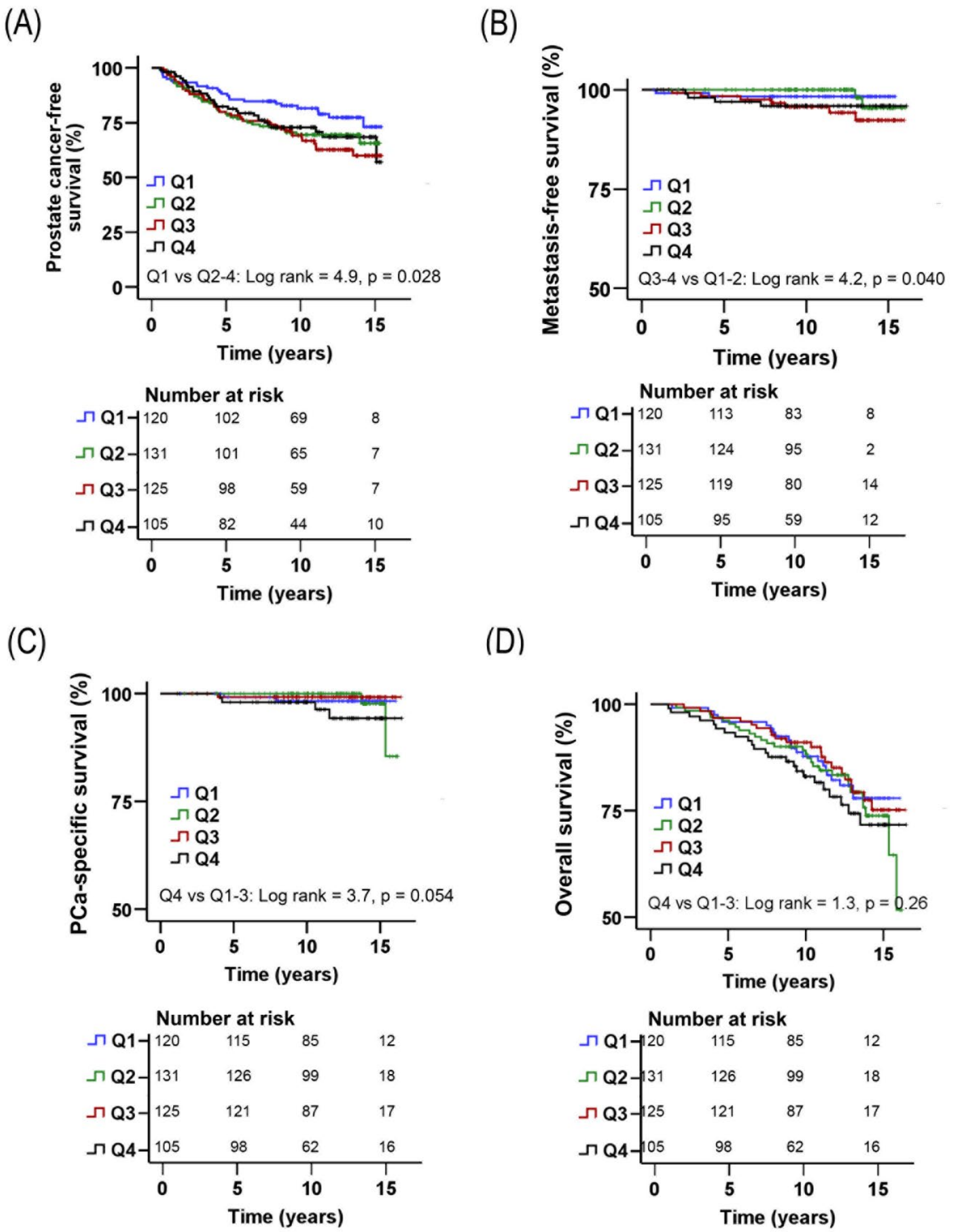

Fig. S3. Pre-diagnostic plasma pro-NPY levels predict time to prostate cancer (PCa) diagnosis and metastatic disease. (A) Plasma pro-NPY levels at initial blood sampling in relation to PCa-free survival, according to Kaplan-Meier analysis. (B-D) Kaplan-Meier analysis of plasma pro-NPY levels in relation to metastasis-free survival (B), PCa-specific survival (C), and overall survival (D). The pro-NPY levels were divided in quartiles (Q1-Q4) based on all samples in the cohort (n=796).
