# Supplementary material for: Pro-neuropeptide Y as a circulating biomarker for poor prognosis in prostate cancer
Source: Sci Rep. 2026 Jun 23;16:19518. doi: 10.1038/s41598-026-58517-8 (PMC13291266; doi:10.1038/s41598-026-58517-8)
Supplement: Supplementary file 4 — Supplementary Information 4. [file 41598_2026_58517_MOESM4_ESM.pdf]

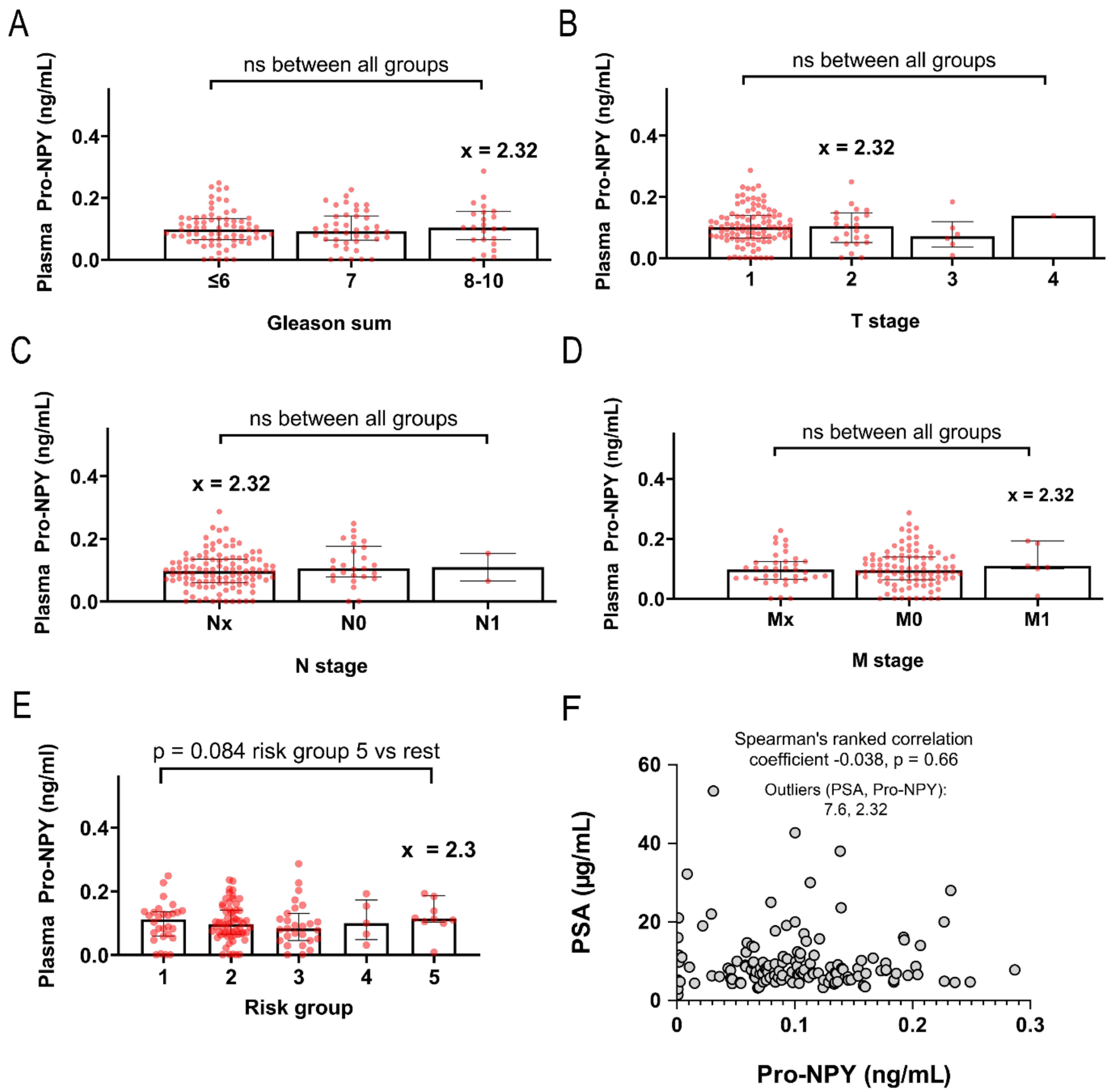

Fig. S4. Pre-diagnostic plasma pro-NPY levels in patients diagnosed with prostate cancer during follow-up (n=137, cohort 1) in relation to Gleason score (A), T stage (B), N stage (C), M stage (D), risk group (E) and plasma PSA (F) at diagnosis (Table S1-2). Bars show median and inter-quartile range with individual values plotted. *P <0.05 according to Mann-Whitney U test. x = outlier value (ng/mL). Bivariate correlation between plasma pro-NPY and serum PSA was assessed with Spearman's ranked correlation coefficient.
