# Supplementary material for: Pro-neuropeptide Y as a circulating biomarker for poor prognosis in prostate cancer
Source: Sci Rep. 2026 Jun 23;16:19518. doi: 10.1038/s41598-026-58517-8 (PMC13291266; doi:10.1038/s41598-026-58517-8)
Supplement: Supplementary file 5 — Supplementary Information 5. [file 41598_2026_58517_MOESM5_ESM.pdf]

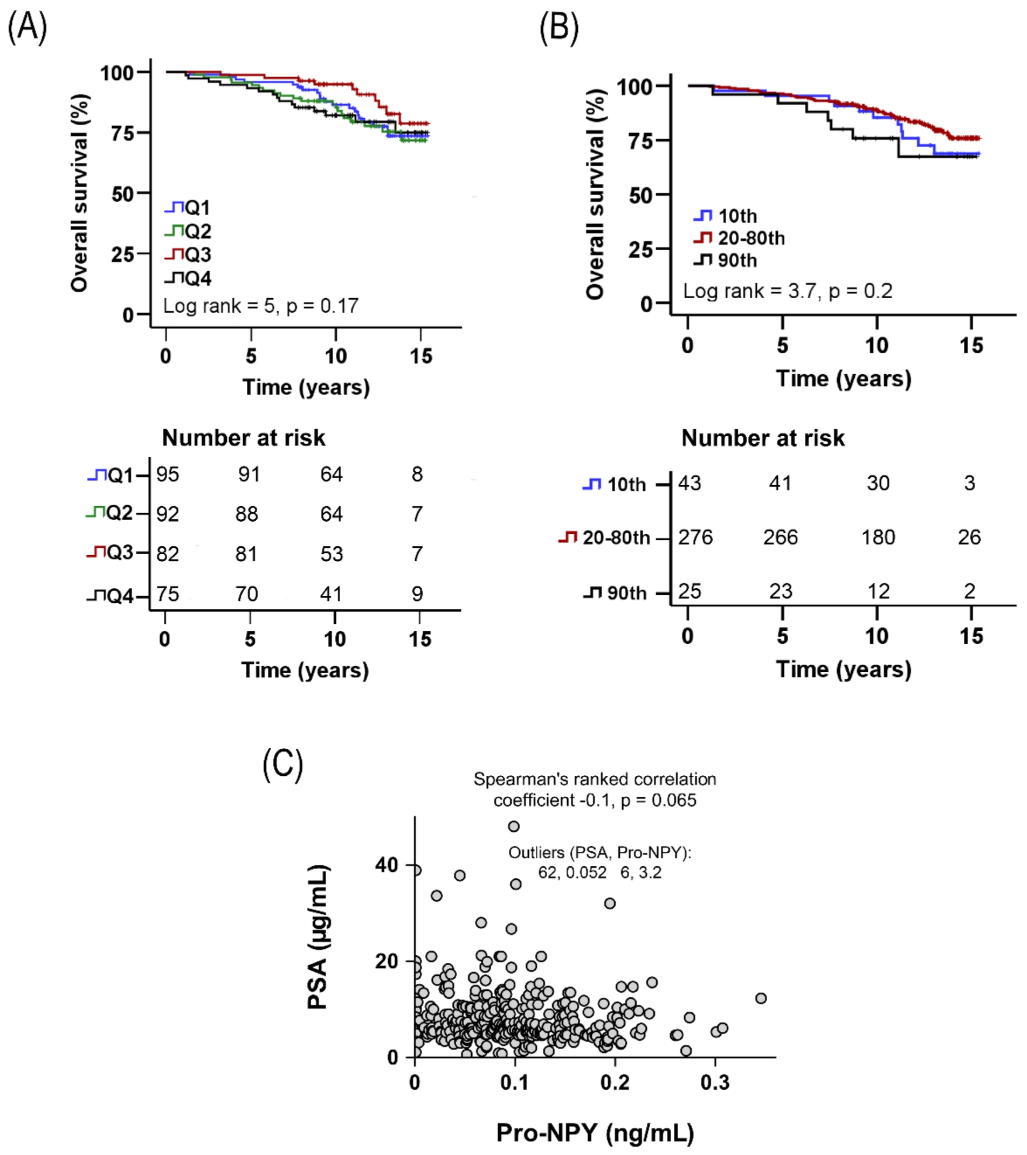

Fig. S5. Plasma pro-NPY in relation to overall survival and serum PSA in patients who remained disease-free during follow-up in cohort 1. (A-B) Kaplan Meier survival analysis with patients divided in 4 groups based on quartile levels (A), and 3 groups using cut-off values separating the 10th and 90th percentiles (B). Subgrouping was based on all cohort samples (n = 796) and the Log rank test was performed to compare survival distributions. (C) Bivariate correlation between plasma pro-NPY and serum PSA, assessed by Spearman's ranked correlation coefficient.
