# Supplementary material for: Pro-neuropeptide Y as a circulating biomarker for poor prognosis in prostate cancer
Source: Sci Rep. 2026 Jun 23;16:19518. doi: 10.1038/s41598-026-58517-8 (PMC13291266; doi:10.1038/s41598-026-58517-8)
Supplement: Supplementary file 6 — Supplementary Information 6. [file 41598_2026_58517_MOESM6_ESM.pdf]

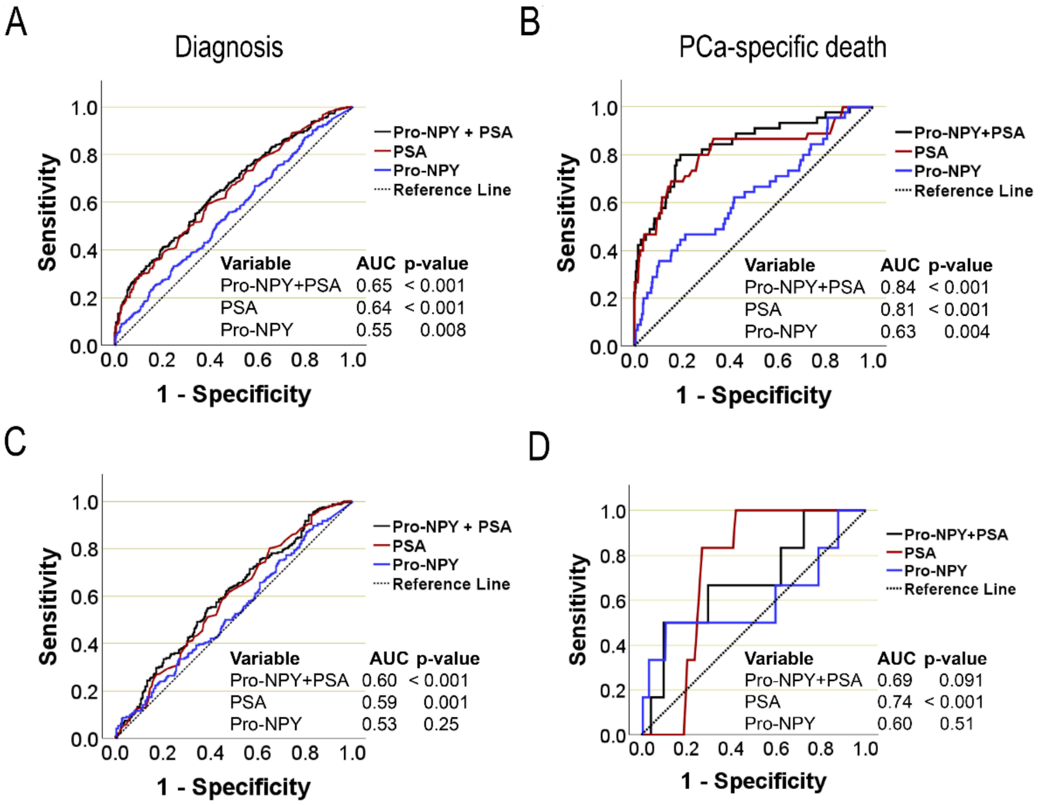

Fig. S6. Plasma pro-NPY independently and in combination with serum PSA predicts PCa-diagnosis and PCa-specific death within 10 years from blood sampling. ROC analysis of plasma pro-NPY (blue), PSA (red) and both markers combined (black) in relation to their ability to predict PCa diagnosis (A, C) and PCa-specific death (B, D). Time to PCa-diagnosis was explored based on all patients (A, 437 events among 796 patients) or cases with PSA levels <10 µg/l (C, 242 events among 503 patients). PCa-specific death was assessed in all patients with adequate follow-up time (B, 45 events among 544 patients) and in cases with PSA levels <10 µg/l (D, 6 events among 339 patients). The combined model was created using a binary logistic regression and all graphs constructed with logarithmized dependent variables (log10). Mann-Whitney U test was used to check if model was significantly different from the reference line. Delong's test was applied to compare the AUC of different models.
