# Supplementary material for: Pro-neuropeptide Y as a circulating biomarker for poor prognosis in prostate cancer
Source: Sci Rep. 2026 Jun 23;16:19518. doi: 10.1038/s41598-026-58517-8 (PMC13291266; doi:10.1038/s41598-026-58517-8)
Supplement: Supplementary file 7 — Supplementary Information 7. [file 41598_2026_58517_MOESM7_ESM.pdf]

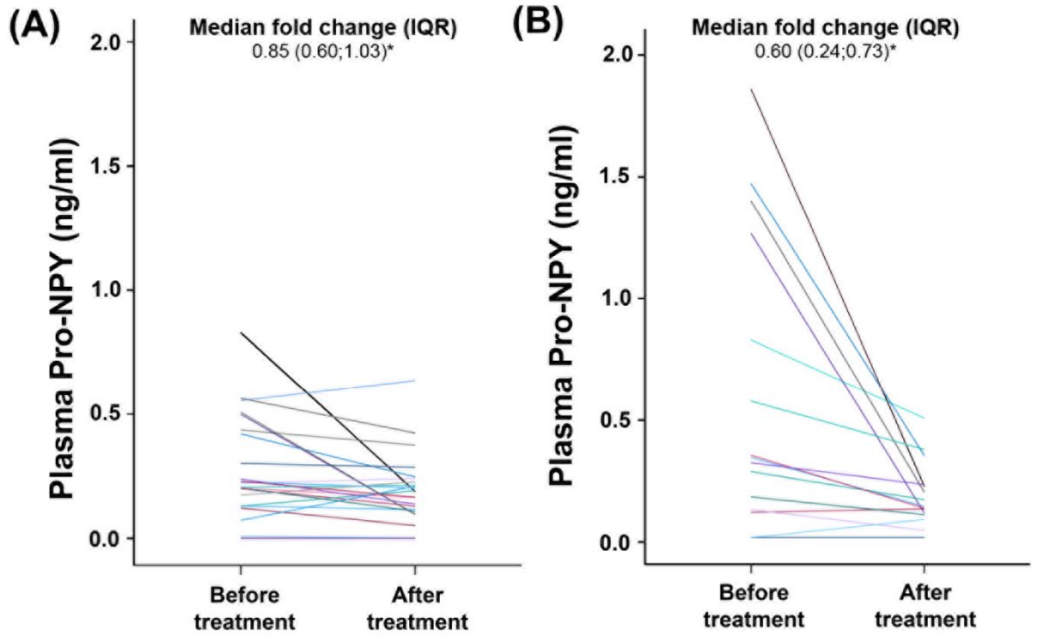

Fig. S7. Plasma pro-NPY levels are reduced after androgen-deprivation therapy (ADT). Plasma pro-NPY levels in individual patients before and about 3 months after initiation of primary treatment with A) Neoadjuvant ADT given prior to radiation therapy, and B) continuous ADT. Median fold change and inter-quartile range (IQR) per treatment group are given. *$p < 0.05$, according to Wilcoxon signed rank test for paired observations (pre- and post-treatment levels in the same individual are indicated by lines).
