# Supplementary material for: Pro-neuropeptide Y as a circulating biomarker for poor prognosis in prostate cancer
Source: Sci Rep. 2026 Jun 23;16:19518. doi: 10.1038/s41598-026-58517-8 (PMC13291266; doi:10.1038/s41598-026-58517-8)
Supplement: Supplementary file 8 — Supplementary Information 8. [file 41598_2026_58517_MOESM8_ESM.pdf]

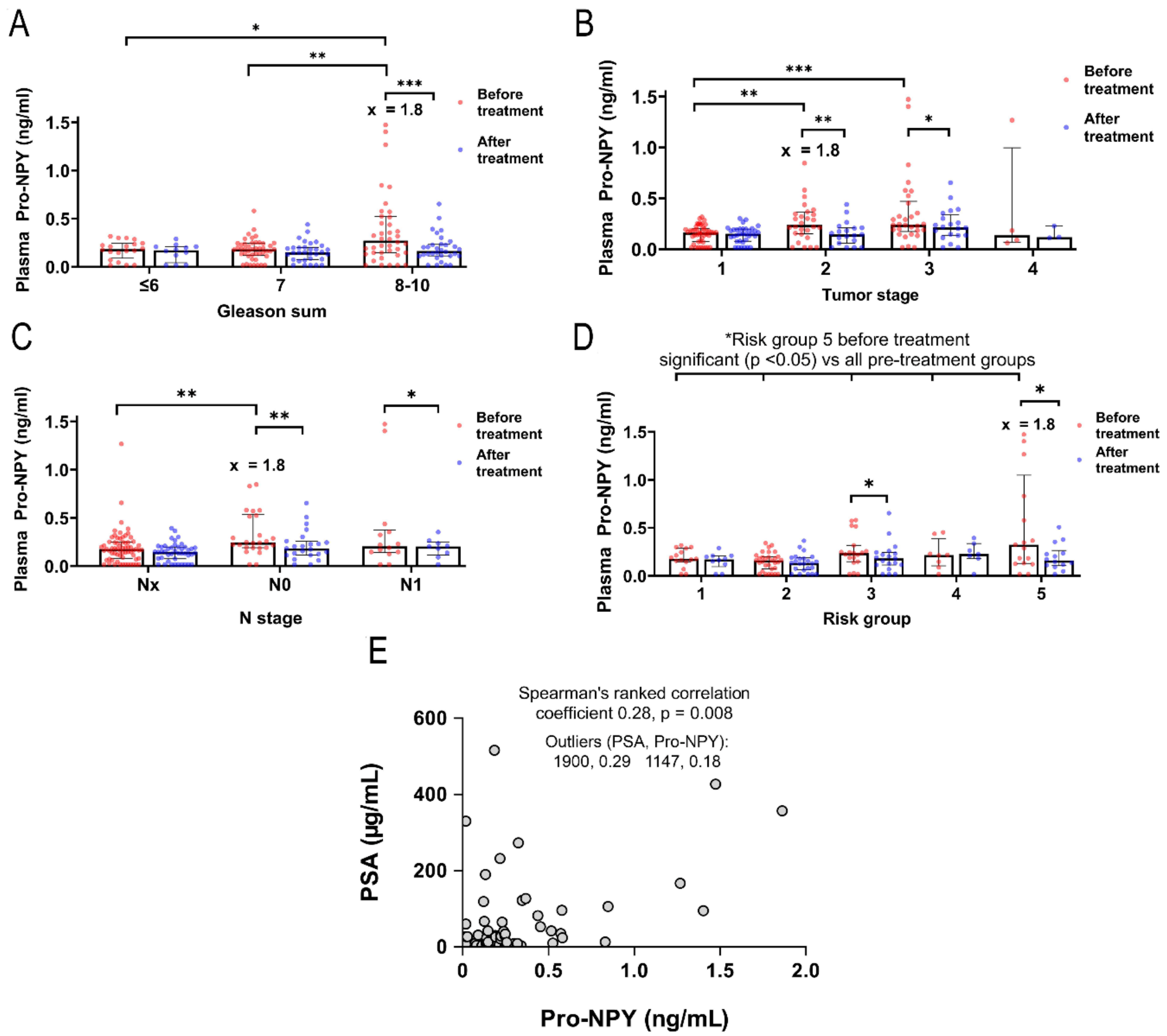

Fig. S8. Plasma pro-NPY in relation to clinical parameters for patients in cohort 2. (A-D) Plasma levels before and about 3 months after treatment initiation subdivided by Gleason score (A), T stage (B), N stage (C) and risk group (D) (Table S1-2). Bars show median and inter-quartile range with individual values plotted. $^*p < 0.05$, $^{**}p < 0.01$, $^{***}p < 0.001$, respectively, according to Mann-Whitney U test comparing pre-treatment levels and Wilcoxon for pre- vs post treatment comparisons. x = outlier values (ng/ml). (E) Bivariate correlation analysis between plasma pro-NPY and serum PSA, using Spearman's ranked correlation coefficient.
