# Supplementary material for: Pro-neuropeptide Y as a circulating biomarker for poor prognosis in prostate cancer
Source: Sci Rep. 2026 Jun 23;16:19518. doi: 10.1038/s41598-026-58517-8 (PMC13291266; doi:10.1038/s41598-026-58517-8)
Supplement: Supplementary file 9 — Supplementary Information 9. [file 41598_2026_58517_MOESM9_ESM.pdf]

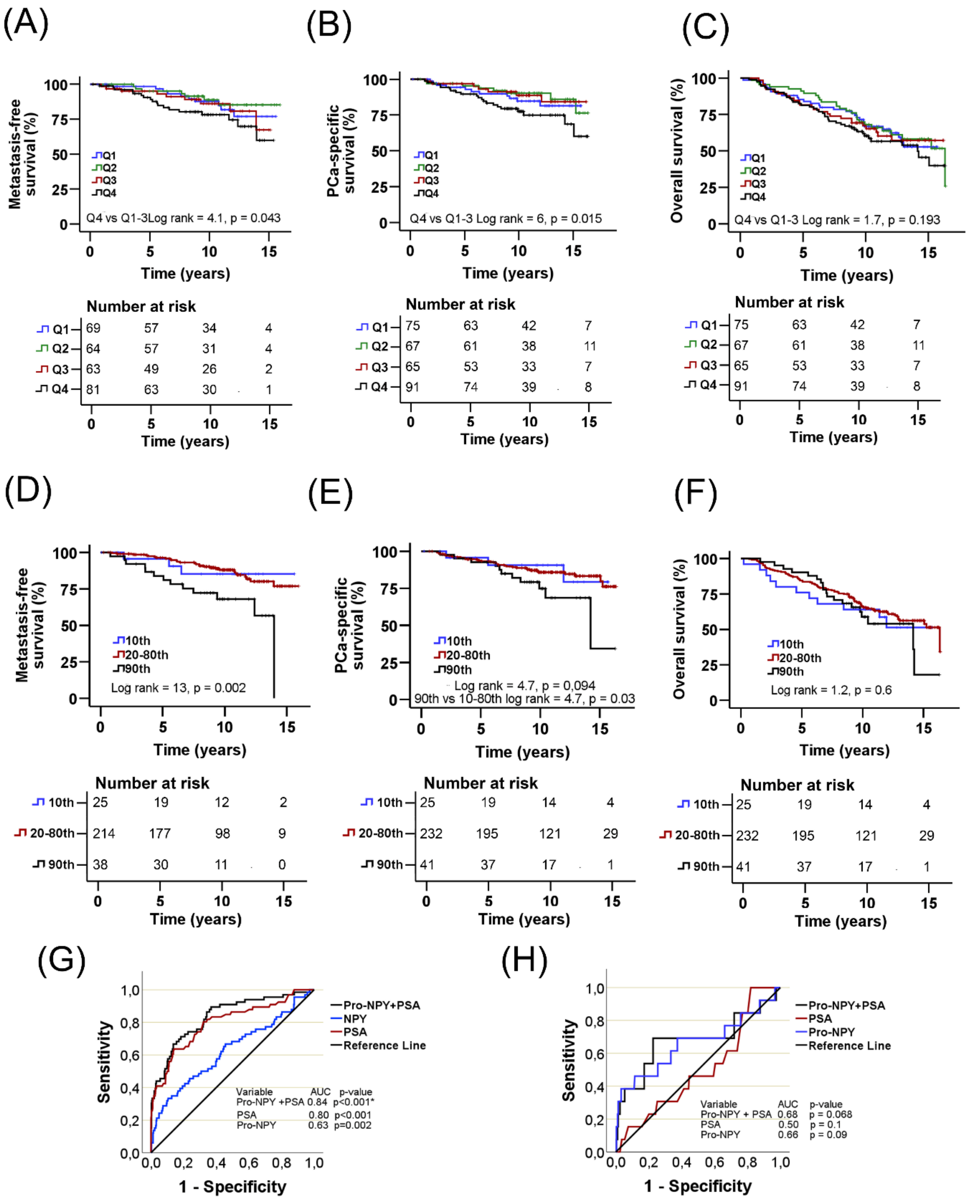

Fig. S9. Sensitivity analysis by exclusion of patients in cohort 1 with circulating pro-NPY levels below the lower limit of quantification (LLOQ). (A-F) Kaplan-Meier analysis of plasma pro-NPY levels in patients diagnosed at sampling in relation to metastasis-free survival (A, D), PCa-specific survival (B, E), and overall survival (C, F). The pro-NPY levels were analyzed in quartiles (Q1-Q4) in A-C and in 3 groups cut by the 10th and 90th percentiles in D-F, based on samples above the LLOQ (n = 740). Patients with metastasis diagnosis at the time for blood sampling were excluded in analysis of metastasis-free survival (A, D). (G-H) Plasma pro-NPY independently and in combination with serum PSA predicts metastasis within 10 years from blood sampling after exclusion of patients with pro-NPY levels below the lower limit of quantification (LLOQ). ROC analysis of plasma pro-NPY (blue), PSA (red)

and both markers combined (black) in relation to their ability to predict metastasis within 10 years in all patients with adequate follow-up (G, 66 events among 477 patients) and in cases with PSA levels <10 μg/l (H, 13 events among 291 patients). Combined models were created using binary logistic regression and graphs constructed with logarithmized dependent variables (log10). Mann-Whitney U test was used to check if model was significantly different from the reference line. Delong's test was applied to compare the AUC of different models. *$P < 0.05$ when comparing the combined model with the PSA model.
