# Supplementary material for: Pro-neuropeptide Y as a circulating biomarker for poor prognosis in prostate cancer
Source: Sci Rep. 2026 Jun 23;16:19518. doi: 10.1038/s41598-026-58517-8 (PMC13291266; doi:10.1038/s41598-026-58517-8)
Supplement: Supplementary file 10 — Supplementary Information 10. [file 41598_2026_58517_MOESM10_ESM.pdf]

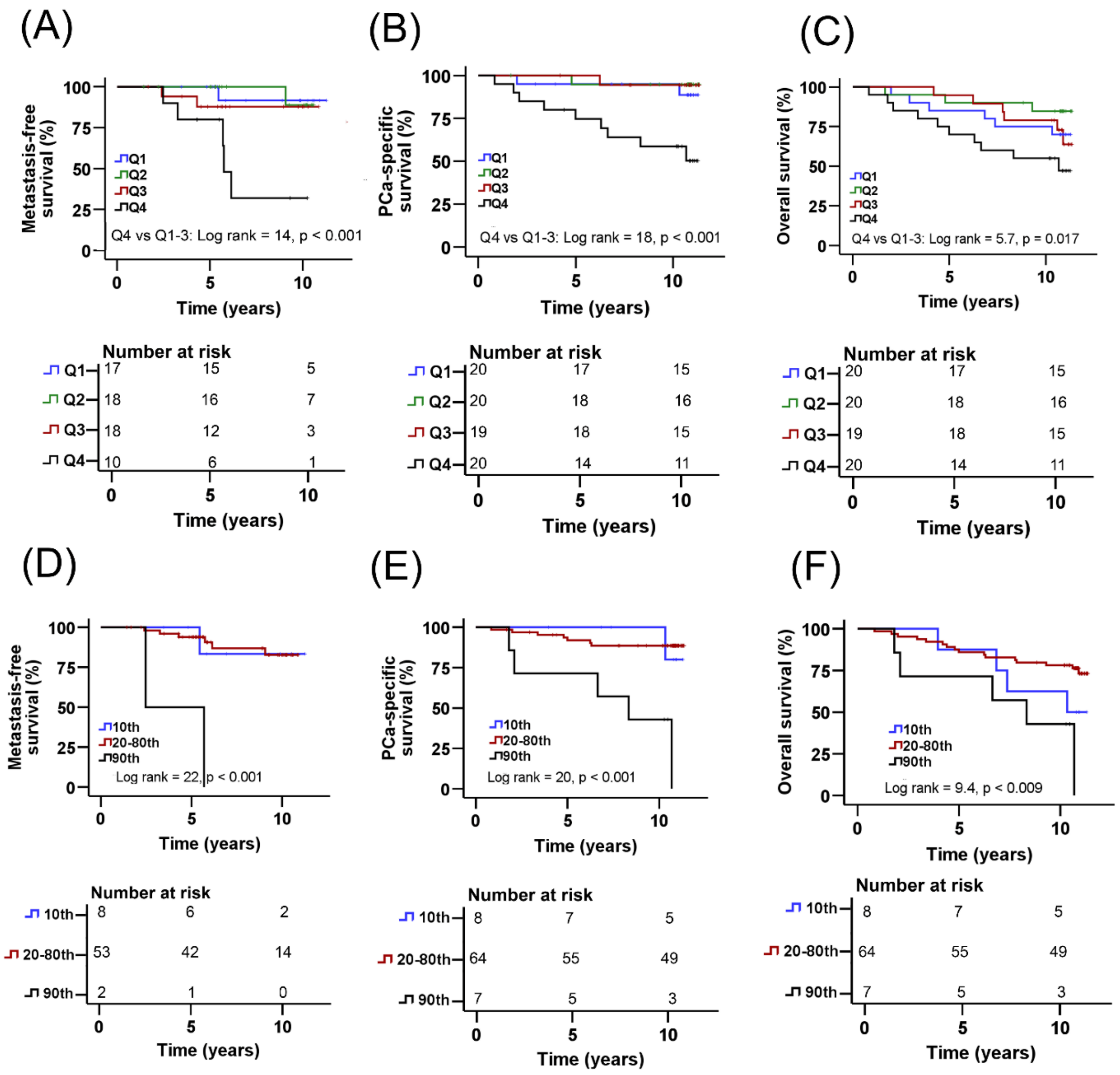

Fig. S10. Sensitivity analysis by exclusion of patients in cohort 2 with circulating pro-NPY levels below the lower limit of quantification (LLOQ). (A-F) Kaplan Meier analysis of plasma pro-NPY levels before treatment in relation to metastasis-free survival (A, D), PCa-specific survival (B, E) and overall survival (C, F). Patients were divided into quartiles (Q1-Q4) in A-B and in 3 groups based on the 10th and 90th percentiles in D-F based on pre-treatment pro-NPY samples levels above the LLOQ (n = 79). Patients with metastasis diagnosis at the time for blood sampling were excluded in analysis of metastasis-free survival (A, D).
